# Mobile genetic elements drive the evolution and multidrug resistance of *Salmonella* infantis along the United States poultry production line

**DOI:** 10.1186/s12864-026-12607-z

**Published:** 2026-03-03

**Authors:** Yujie Zhang, Mackenna Chu, Sanghvi Samala, Alexandra Salvador, Yen-Te Liao, Vivian C.H. Wu

**Affiliations:** Produce Safety and Microbiology Research Unit, U.S. Department of Agriculture, Agricultural Research Service, Western Regional Research Center, Albany, CA 94710 USA

**Keywords:** *Salmonella* infantis, Poultry production line, Whole genome sequencing, Mega-plasmid, Mobile genetic elements, Multiple-drug resistance

## Abstract

**Background:**

*Salmonella* is the primary enteric pathogen related to foodborne illnesses worldwide, posing significant public health concerns. Amongst the diversity and pathogenicity of over 2000 *Salmonella* serovars, *Salmonella* Infantis (*S.* Infantis) ranks among the top *Salmonella* serovars implicated in foodborne outbreaks, with thousands of reported cases annually in the United States. Moreover, the incidence of *S.* Infantis infections has spread rapidly worldwide from the U.S. to Europe, where more than 50% of isolated *S.* Infantis strains have developed antibiotic resistance. Previous studies have demonstrated that antibiotic resistance genes (ARGs), particularly those carried by plasmids, contribute to the persistence and spread of multipledrug-resistant (MDR) *Salmonella* strains in various environments. However, the information regarding the multidrug resistance and spread of *S.* Infantis is scarce. Hence, the objectives of this study were to characterize antibiotic-resistant *S.* Infantis isolated from the poultry production line in the United States and to examine the correlation between mobile genetic elements and bacterial resistome evolution.

**Results:**

A total of 9 *S.* Infantis strains were isolated from poultry production lines in 2022, including comminuted chicken, raw intact/nonintact chicken, and chicken carcass. These strains were further subject to antimicrobial susceptibility tests, whole-genome sequencing, and bioinformatic analysis. Most strains were MDR and phenotypically resistant to five or more antibiotics, such as Streptomycin and Tetracycline. The complete bacterial genome results showed that all isolates had a dsDNA chromosome with a GC content of 52.3% and an average genome size of 4,730 kb, belonging to the sequence type 32. The genomic characterization of *S.* Infantis isolates revealed that each strain contained one IncFIB mega-plasmid with lengths from 289 to 327 kb. Five or more ARGs were detected in each strain, most of which were located in the mega-plasmids and bordered by diverse mobile genetic elements, including transposons, integrons, and prophages. The common ARGs present in the mega-plasmids included *bla*_CTX−M−65_, *aac(3)-IV*, *tetA*, *suil1*, *dfrA14*, *ant(3’’)-Ia* and *floR.* Moreover, the majority of whole-genome sequencing (WGS)-derived ARG profiles had concordant phenotypic traits.

**Conclusion:**

These findings reveal the genomic features and antimicrobial resistance profiles of *S.* Infantis strains from poultry production lines in the United States, indicating the potential of mobile genetic elements-driven *S.* Infantis resistome development.

**Supplementary Information:**

The online version contains supplementary material available at 10.1186/s12864-026-12607-z.

## Data summary

All supporting data, code, and protocols have been provided within the article or through supplementary data files. One supplementary figure is available in the online version of this article.

## Background

*Salmonella* is a primary enteric pathogen related to foodborne illnesses worldwide, with over 3 million deaths, posing significant public health concerns [1, 2]. Amongst 2659 *Salmonella* serovars, Centers for Disease Control and Prevention (CDC) reported several *Salmonella* serovars commonly found in poultry products –Enteritidis, Infantis, and Typhimurium—were highly associated with foodborne illnesses [3]. Additionally, these three serovars have been identified as highly virulent Key Performance Indicators (KPI) in poultry products by the United States Department of Agriculture -Food Safety and Inspection Service (USDA-FSIS) for the 2022–2026 years [3]. Particularly, *S.* Infantis ranks among the top most common *Salmonella* serovars implicated in foodborne outbreaks originating from poultry sources; the CDC has recently designated this pathogen as a REP (recurring, emerging, and persistent) strain, with thousands of reported cases annually in the United States alone [4]. The bacterium is known to colonize the gastrointestinal tract of poultry due to inadequate hygienic practices, leading to the contamination of meat and eggs. Furthermore, the pathogen can be transmitted to humans through the consumption of undercooked poultry products or cross-contamination during food preparation [5, 6].

The use of antibiotics has been a common practice in treating salmonellosis and preventing further bacterial growth; however, there has been a rising concern regarding the increase in antimicrobial resistance (AMR) observed in *S.* Infantis strains on a global scale [7]. Specifically, *S.* Infantis strains, commonly associated with poultry products, have exhibited alarming resistance to antibiotics, including fluoroquinolones, cephalosporins, and ampicillin, which are crucial for treating human diseases [8]. The emergence and spread of antimicrobial resistance in *S.* Infantis can be attributed to various factors, such as the indiscriminate use of antibiotics in animal agriculture and inadequate hygiene practices along the food production chain. According to recent studies, the prevalence of *S.* Infantis has a unique characteristic that allows it to gain AMR genes, which can further spread and influence other *Salmonella* strains through environmental means. This feature of *S.* Infantis requires more attention and research focus to mitigate its global impact [9, 10].

In response to the growing threat of AMR in *S.* Infantis, researchers have explored methods and tools to alleviate public health concerns. There have been suggestions to use a wider range of vaccines and probiotics to minimize the usage of antibiotics. However, these strategies do not solve the general issue regarding antimicrobial resistance and its origin [9]. Identifying, targeting, and preventing AMR through molecular and epidemiological means will allow for a more precise understanding of the correlation between AMR and the origin of resistant bacterial transmission [11]. For example, whole-genome sequencing (WGS) and genomic characterization are indispensable for understanding the emergence and spread of multidrug-resistant (MDR) *S.* Infantis strains, which showed resistance to three or more antibiotic classes [12, 13]. These techniques enable accurate tracking of transmission pathways, identification of AMR mechanisms, and differentiation of clonal lineages, thus enhancing epidemiological surveillance and outbreak investigations [14]. Previous studies showed that specific genetic markers and resistance genes, such as those carried on by plasmids, contributed to the persistence and spread of MDR in *S.* Infantis strains in various environments, especially in poultry production [15, 16]. Additionally, WGS facilitates the specific prediction of resistance profiles, informing targeted public health interventions and optimizing clinical treatments [17]. These insights are critical for developing effective control measures to mitigate the public health impact of MDR *Salmonella* Infantis [18]. The ongoing battle against *Salmonella* Infantis AMR/MDR warrants a comprehensive study to elucidate its epidemiology, transmission dynamics, and antimicrobial resistance profiles. Therefore, this study aims to identify and understand the intricacies of *S.* Infantis. These results will illuminate its importance as a pathogen of concern and the imperative need for continued research in this field.

## Methods

### Isolation and antimicrobial susceptibility testing of *S.* Infantis strains

A total of 9 *S.* Infantis strains were obtained from USDA-FSIS and further subjected to antimicrobial susceptibility testing and whole-genome sequencing in this study. These strains were previously isolated from the United States chicken production line in 2022 by USDA-FSIS routine screenings (Table 1). The antimicrobial susceptibility testing of 9 *S.* Infantis strains was conducted using the disc diffusion method on Muller-Hinton agar (MHA) (Oxoid, USA) according to Clinical and Laboratory Standards Institute (CLSI) recommendations [18]. A total of twenty-six antimicrobial susceptibility discs (Oxoid™, USA) from different classes were used for the test (Table 2). The size of the inhibition zone caused by the antibiotic discs was measured to determine bacterial resistance based on CLSI standards.

**Table 1:** Overview of 9 *Salmonella* infantis strains isolated from the poultry production line in the united States

Strain ID	Location	Year	Isolation source	Serovar	Predicted antigenic profile	MLST	Chromosome (bp)	Plasmid (bp)	GC content
FSIS12214897	USA: NY	2022	chicken carcass	Infantis	7:r:1,5	ST32	4,726,701	286,902; 33,657	52.3%
FSIS12214900	USA: GA	2022	comminuted chicken	Infantis	7:r:1,5	ST32	4,727,115	327,150	52.3%
FSIS12215221	USA: AR	2022	comminuted turkey	Infantis	7:r:1,5	ST32	4,728,609	319,153	52.3%
FSIS22209799	USA: CA	2022	raw intact chicken	Infantis	7:r:1,5	ST32	4,727,083	310,289	52.3%
FSIS22209851	USA: WI	2022	comminuted chicken	Infantis	7:r:1,5	ST32	4,733,548	303,375	52.3%
FSIS22209861	USA: CA	2022	comminuted chicken	Infantis	7:r:1,5	ST32	4,731,576	319,112	52.3%
FSIS22209916	USA: AR	2022	raw intact chicken	Infantis	7:r:1,5	ST32	4,736,541	319,200	52.3%
FSIS32207821	USA: AR	2022	nonintact chicken	Infantis	7:r:1,5	ST32	4,725,739	310,270	52.3%
FSIS32207823	USA: TX	2022	chicken - young chicken carcass rinse (post-chill)	Infantis	7:r:1,5	ST32	4,727,410	295,501	52.3%

**Table 2:** Correlation of phenotypic resistance with ARG profiles of 9 S. Infantis isolates

Drug Class	Isolate	*S.* Infantis 9851		*S.* Infantis 7821		*S.* Infantis 4900		*S.* Infantis 5221		*S.* Infantis 9861		*S.* Infantis 9799		*S.* Infantis 9916		*S.* Infantis 4897		*S.* Infantis 7823	
	Drug	AMR	Resfinder	AMR	Resfinder	AMR	Resfinder	AMR	Resfinder	AMR	Resfinder	AMR	Resfinder	AMR	Resfinder	AMR	Resfinder	AMR	Resfinder
Fluoroquinolones	Moxifloxacin (5 ug)	S	*/*	S	*/*	S	*/*	S	*/*	S	*/*	S	*/*	S	*/*	S	*/*	S	*/*
	Ciprofloxacin (5 ug)	I	*/*	I	*/*	I	*/*	I	*/*	I	*/*	I	*/*	I	*/*	I	*/*	I	*/*
Cephalosporins	Ceftazidimine (30 ug)	S	*/*	S	***bla***_**CTX−M−65**_	S	***bla***_**CTX−M−65**_	S	***bla***_**CTX−M−65**_	S	***bla***_**CTX−M−65**_	S	***bla***_**CTX−M−65**_	S	***bla***_**CTX−M−65**_	S	*/*	S	***bla***_**CTX−M−65**_
	Cefoperazone (75 ug)	S	*/*	R	*/*	R	*/*	R	*/*	R	*/*	R	*/*	R	*/*	S	*/*	R	*/*
	Cefoxitin (30 ug)	S	*/*	S	*/*	S	*/*	S	*/*	S	*/*	S	*/*	S	*/*	S	*/*	S	*/*
	Cefotaxime (30 ug)	I	*/*	R	***bla***_**CTX−M−65**_	R	***bla***_**CTX−M−65**_	R	***bla***_**CTX−M−65**_	R	***bla***_**CTX−M−65**_	R	***bla***_**CTX−M−65**_	R	***bla***_**CTX−M−65**_	I	*/*	R	***bla***_**CTX−M−65**_
	Ceftriaxone (30 ug)	S	*/*	R	***bla***_**CTX−M−65**_	R	***bla***_**CTX−M−65**_	R	***bla***_**CTX−M−65**_	R	***bla***_**CTX−M−65**_	R	***bla***_**CTX−M−65**_	R	***bla***_**CTX−M−65**_	S	*/*	S	***bla***_**CTX−M−65**_
	Cefepime (30 ug)	S	*/*	SDD	***bla***_**CTX−M−65**_	SDD	***bla***_**CTX−M−65**_	R	***bla***_**CTX−M−65**_	SDD	***bla***_**CTX−M−65**_	SDD	***bla***_**CTX−M−65**_	SDD	***bla***_**CTX−M−65**_	S	*/*	SDD	***bla***_**CTX−M−65**_
	Cefazolin (30 ug)	S	*/*	R	*/*	R	*/*	R	*/*	R	*/*	R	*/*	R	*/*	S	*/*	R	*/*
Aminoglycosides	Streptomycin (10 ug)	R	***ant(3’’)-Ia***	R	***ant(3’’)-Ia***	I	***ant(3’’)-Ia***	R	***ant(3’’)-Ia***	R	***ant(3’’)-Ia***	R	***ant(3’’)-Ia***	R	***ant(3’’)-Ia***	R	***ant(3’’)-Ia***	R	***ant(3’’)-Ia***
	Amikacin (30 ug)	S	*aac(6’)-Iaa*	S	*aac(6’)-Iaa*	S	*aac(6’)-Iaa*	S	*aac(6’)-Iaa*	I	*aac(6’)-Iaa*	S	*aac(6’)-Iaa*	S	*aac(6’)-Iaa*	I	*aac(6’)-Iaa*	S	*aac(6’)-Iaa*
	Kanamycin (30 ug)	S	*/*	S	*/*	R	*/*	S		R	*/*	R	*/*	S	*/*	S	*/*	S	*/*
	Gentamicin (10 ug)	R	***aac(3)-IVa***	R	***aac(3)-IVa***	R	***aac(3)-IVa***	R	***aac(3)-IVa***	R	***aac(3)-IVa***	R	***aac(3)-IVa***	S	***aac(3)-IVa***	S	*/*	R	***aac(3)-IVa***
	Tobramycin (10 ug)	R	*/*	R	***bla***_**CTX−M−65**_	R	***bla***_**CTX−M−65**_	R	***bla***_**CTX−M−65**_	R	***bla***_**CTX−M−65**_	R	***bla***_**CTX−M−65**_	S	***bla***_**CTX−M−65**_	S	*/*	R	***bla***_**CTX−M−65**_
Carbapenems	Meropenem (10 ug)	S	*/*	S	*/*	S	*/*	S	*/*	S	*/*	S	*/*	S	*/*	S	*/*	S	*/*
	Imipenem (10 ug)	S	*/*	S	*/*	S	*/*	S	*/*	S	*/*	S	*/*	S	*/*	S	*/*	S	*/*
Penicillins	Piperacillin (100 ug)	S	*/*	R	***bla***_**CTX−M−65**_	R	***bla***_**CTX−M−65**_	R	***bla***_**CTX−M−65**_	R	***bla***_**CTX−M−65**_	R	***bla***_**CTX−M−65**_	R	***bla***_**CTX−M−65**_	S	*/*	R	***bla***_**CTX−M−65**_
	Ampicillin (10 ug)	S	*/*	R	***bla***_**CTX−M−65**_	R	***bla***_**CTX−M−65**_	R	***bla***_**CTX−M−65**_	R	***bla***_**CTX−M−65**_	R	***bla***_**CTX−M−65**_	R	***bla***_**CTX−M−65**_	I	*/*	R	***bla***_**CTX−M−65**_
	Ticarcillin (75 ug)	S	*aac(6’)-Iaa*, ***aac(3)-IVa***	R	*aac(6’)-Iaa*, ***aac(3)-IVa***	R	*aac(6’)-Iaa*, ***aac(3)-IVa***	R	*aac(6’)-Iaa*	R	*aac(6’)-Iaa*, ***aac(3)-IVa***	R	*aac(6’)-Iaa*, ***aac(3)-IVa***	R	*aac(6’)-Iaa*, ***aac(3)-IVa***	S	*aac(6’)-Iaa*	R	*aac(6’)-Iaa*, ***aac(3)-IVa***
B-Lactam Combination Agents	Amoxicillin-clavulanate (30 ug)	S	*/*	S	*/*	S	*/*	S	*/*	S	*/*	S	*/*	S	*/*	S	*/*	R	*/*
	Ampicillin-sulbactam (20 ug)	S	*/*	S	*/*	S	*/*	S	*/*	S	*/*	S	*/*	S	*/*	S	*/*	S	*/*
Tetracyclines	Tetracycline (30 ug)	R	***tet(A)***	R	***tet(A)***	R	***tet(A)***	R	***tet(A)***	R	***tet(A)***	R	***tet(A)***	R	***tet(A)***	R	***tet(A)***	I	***tet(A)***
	Doxyxycline (30 ug)	I	*/*	I	***bla***_**CTX−M−65**_	I	***bla***_**CTX−M−65**_	I	***bla***_**CTX−M−65**_	I	***bla***_**CTX−M−65**_	R	***bla***_**CTX−M−65**_	I	***bla***_**CTX−M−65**_	I	*/*	I	***bla***_**CTX−M−65**_
Phenicols	Chloramphenicol (30 ug)	R	***floR***	S	***floR***	R	***floR***	R	***floR***	R	***floR***	R	***floR***	S	***floR***	S	*/*	R	***floR***
Folate Pathway Antagonists	Trimethoprim-sulfamethoxazole (25 ug)	S	***sul1***	S	***sul1***	R	***dfrA14***, ***sul1***	S	***dfrA14***	R	***dfrA14***, ***sul1***	R	***sul1***	S	***sul1***,***dfrA14***	S	***sul1***	S	***sul1***
Monobactams	Aztreonam (30 ug)	S	***tet(A)***	R	***tet(A)***	R	***tet(A)***	R	***tet(A)***	R	***tet(A)***	R	***tet(A)***	R	***tet(A)***	S	***tet(A)***	R	***tet(A)***

_S: Susceptible; SDD: Susceptible Dose−Dependent; I: Intermediate; R: Resistant; /: there are no corresponding ARGs present in the strain; Bold font indicates genes located in plasmid contigs_

### DNA extraction and whole genome sequencing

Genomic DNA was extracted from bacterial cultures grown to mid-exponential phase in 10 ml tryptic soy broth (TSB; Difco, Becton, Dickinson, Sparks, MD) using a Promega Wizard^®^ HMW DNA Extraction Kit (Promega, Madison, USA) according to the manufacturer’s instructions. DNA was quantified using Qubit and a Nanodrop 8000, following the manufacturer’s instructions. The DNA library was constructed using Native Barcoding Kit 24 V14 (SQK-NBD114.24, Oxford Nanopore Technologies, Oxford, UK). The prepared library was subsequently loaded onto a FLO-MIN114 Flow Cell (R10.4.1) (Oxford Nanopore Technologies) and sequenced via the MinION Mk1C sequencer for 48–72 h with the default settings. Base calling and quality control were performed in real-time by MinKNOW with dorado/0.4.2 (Oxford Nanopore Technologies).

### Bioinformatic analysis

Raw FASTQ reads of each strain were subjected to adapter trimming and correction using Porechop (Version 0.2.4) and Canu (Version 2.2), with the default settings. The corrected reads were assembled using Flye (Version 2.9.1). The resulting circular contigs were identified as complete chromosomes and plasmids using Busco 5 (version 5.4.5) and BLASTn, respectively. The complete genomes were annotated using the National Center for Biotechnology Information (NCBI) Prokaryotic Genome Annotation Pipeline (PGAP, Version 1.14.6). Core genome multi-locus sequence typing (cgMLST) of nine bacterial chromosomes was conducted using cgMLSTFinder 1.2 (https://cge.food.dtu.dk/services/cgMLSTFinder/, accessed on 04/17/2024) and visualized via Interactive Tree Of Life (iTOL v6) [19]. The classification of plasmid type was identified using PlasmidFinder 2.1, with settings of 95% identity and 80% coverage. The prediction of serovar, prophages, virulence genes, antibiotic resistance genes, and mobile genetic elements was performed via SeqSero 1.2, PHASTER, ABRicate, and BacAnt v3.3.1, respectively [20–22]. *Salmonella* pathogenicity islands of each bacterial genome were analyzed via SPIFinder 2.0 [23]. Default parameters were used for all the software unless otherwise specified.

## Results

### Phenotypic resistance profile of *S.* Infantis strains

A total of 9 *S.* Infantis strains were isolated from the poultry production line for regular food safety inspection by USDA-FSIS in the United States (Table 1). The antimicrobial susceptibility testing was conducted to determine their resistance to 26 different antibiotics. Based on the CLSI guidelines, most strains exhibited resistance to five or more antibiotics, including Streptomycin, Gentamicin, Tobramycin, Tetracycline, and Chloramphenicol, indicating they were MDR strains. Only *S.* Infantis 4897 was resistant to two tested antibiotics: Streptomycin and Tetracycline (Table 2). Among these strains, four isolates—*S.* Infantis 4900, 5221, 9861, and 9799—had the most pronounced MDR phenotype and were resistant to 14, 14, 15, and 16 antimicrobials, respectively (Table 2). According to the drug class, most *S.* Infantis strains were resistant to the antibiotics belonging to the categories of Cephalosporins, Aminoglycosides, Penicillin, Tetracyclines, Phenicols, and Monobactams. Additionally, all strains were sensitive to Carbapenems, and only one strain, *S.* Infantis 7823, was resistant to the antibiotic Amoxicillin-clavulanate, which belongs to the drug class B-Lactam Combination Agents. Furthermore, all isolates were subjected to whole-genome sequencing, followed by downstream bioinformatic analysis to investigate their underlying mechanisms of antibiotic resistance.

### Genomic characterization of *S.* Infantis strains

**Fig. 1:**
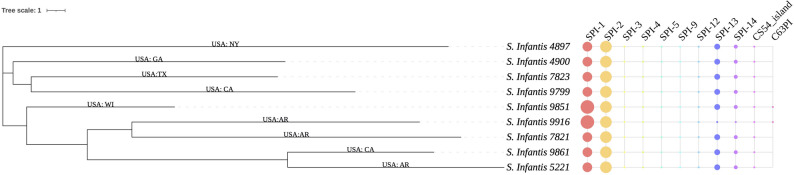
Phylogenic tree of nine *S.* Infantis strains isolated from the poultry production line based on the core-genome MLST. The isolation sources, indicated by geographical location, were listed next to the branches. The presence of *Salmonella pathogenicity* islands (SPI) of each strain was illustrated on the right track. The color and dot sizes correspond to the type and the number of predicted SPI in each strain

Each strain had a double-stranded DNA chromosome, accompanied by one or two plasmids. The chromosomes of these strains exhibited a GC content of 52.3%, and their genome sizes ranged from 4,725,739 to 4,736,541 bp. The genomic analysis of bacterial complete genomes showed that all strains were validated as Infantis serovar and belonged to sequence type (ST) 32 (Table 1). In one of our ongoing studies, the complete genomes of *S.* Infantis from various sources were obtained from the NCBI database. The cgMLST analysis indicated that the nine bacterial strains sequenced in this study were clustered together, differing from other *S.* Infantis isolated from different sources [24]. By digging into unique genomic features, these strains were further classified into three clusters based on cgMLST analysis in this study. Specifically, *S.* Infantis 4897 was classified as cluster 1, while cluster 2 contained *S.* Infantis 4900, *S.* Infantis 7823, and *S.* Infantis 9799. The remaining strains (*n* = 5) fell into cluster 3 (Fig. 1).

### Genomic features of plasmids and prophages

Each strain contained one circular mega-plasmid with a genomic length of 286.9 to 327.1 kb. These mega-plasmids were genomically similar to the plasmid pN55391 (CP016411), which belongs to the IncFIB plasmid group. According to pMLST typing, all IncFIB plasmids belonged to the IncI1 incompatibility group with the nearest ST 71. Four out of five loci of the IncI1 pMLST profile (*ardA*,* pill*,* sogS*, and *trbA*) were found in each IncFIB plasmid, but only *repl1* locus was missing. In addition, *S.* Infantis 4897 also contained a small plasmid with a length of 33,657 bp, classified to the incX1 plasmid.

The result of prophage prediction showed that each *S.* Infantis chromosome contained approximately 5–6 different prophage sequences. These prophages contained genome sizes ranging from 10.2 to 66.8 kb, with GC contents from 46.9% to 53.3%. Among the total of 56 predicted prophages from these 9 bacterial strains, 71.4% of the prophages (*n* = 40), containing most phage functional elements, were intact prophages and able to be induced from the bacterial host, whereas the rest of the prophages (*n* = 16) were questionable. No incomplete prophage was detected in any *S.* Infantis chromosome (Table 3). Furthermore, the most identified prophages (*n* = 11) among all predicted prophages were closely related to PHAGE_Salmon_BTP1 (NC_042346) and were located in the region between 36.0 and 57.2 kb of the bacterial chromosome regardless of the strains. The second most identified prophages (*n* = 10) were similar to PHAGE_Escher_pro483 (NC_028943) and were located in each bacterial genome with a size of around 32.9 kb. The next commonly identified prophage, located in the bacterial host genome between 48.4 and 66.8 kb, was similar to the PHAGE_Salmon_vB_SosS_Oslo (NC_018279). Importantly, these top three prophages were present in the positive bacterial genome as inducible intact prophages. Interestingly, the mega-plasmid within each *S.* Infantis was also detected to contain one or two prophages (Table 3). These prophages were identified as intact prophages, with genome sizes ranging from 12.8 to 33.8 kb and GC content from 47.9% to 51.8%. Different from the prophages’ profile in the bacterial chromosome, the most commonly identified prophages within the plasmids were PHAGE_Escher_RCS47 (*n* = 3, NC_042128), PHAGE_Entero_If1 (*n* = 3, NC_001954), PHAGE_Mycoba_Phrann (*n* = 2, NC_031266), and PHAGE_Mycoba_Squirty (*n* = 2, NC_026588). The phylogenetic analysis of the prophage sequences within bacterial chromosomes and plasmids was further conducted. However, no obvious correlation was observed between the prophage profile and the cgMLST cluster in this study (Figure S1). Prophages within bacterial chromosomes were classified into three distinct clusters, revealing their genomic diversity. Additionally, all prophages carried by plasmids were identified in cluster 3; however, the prophages of *S.* Infantis 9861 and 4900 plasmids were separated from the remaining prophages within other plasmids, falling into a different subcluster of cluster 3.

**Table 3:** Prophages identified in the *S.* Infantis strains of this study

Reference phages	Accession number	No. of isolates	No. of prophages	Category	Location
PHAGE_Burkho_BcepMu	NC_005882	4	4	Intact	Chromosome
PHAGE_Burkho_phiE255	NC_009237	6	6	Intact	Chromosome
PHAGE_Entero_P4	NC_001609	3	3	questionable	Chromosome
PHAGE_Entero_UAB_Phi20	NC_031019	1	1	Intact	Chromosome
PHAGE_Escher_500465_1	NC_049342	7	7	questionable	Chromosome
PHAGE_Escher_pro483	NC_028943	10	10	Intact	Chromosome
PHAGE_Phage_Gifsy_1	NC_010392	3	3	questionable	Chromosome
PHAGE_Salmon_BTP1	NC_042346	10	11	Intact	Chromosome
PHAGE_Salmon_Fels_1	NC_010391	3	3	questionable	Chromosome
PHAGE_Salmon_vB_SosS_Oslo	NC_018279	8	8	Intact	Chromosome
PHAGE_Entero_If1	NC_001954	3	3	Intact	Plasmid
PHAGE_Escher_RCS47	NC_042128	3	3	Intact	Plasmid
PHAGE_Mycoba_Phrann	NC_031266	2	2	Intact	Plasmid
PHAGE_Mycoba_Squirty	NC_026588	2	2	Intact	Plasmid
PHAGE_Salmon_SJ46	NC_031129	1	1	Intact	Plasmid
PHAGE_Shigel_Stx	NC_029120	1	1	Intact	Plasmid

### Virulence factors

Diverse virulence genes, responsible for adherence, antimicrobial activity/competitive advantage, effector delivery system, immune modulation, invasion, nutritional/metabolic factor, and regulation, were detected in each bacterial chromosome). Specifically, most strains contained a total of 258 virulence genes. Among these virulence genes, the majority belonged to the effector delivery system (*n* = 110), including those coding for the type III secretion system, *Salmonella* centrisome island, and type VI secretion system. Moreover, genes related to adherence (*n* = 69) were identified in each bacterial genome, including those encoding fimbrial proteins and curli. Only the chromosome of *S.* Infantis 9851 lacked two virulence genes—*tae4 and hilD*—which encoded the type VI secretion system effector and AraC family transcriptional regulator, respectively; the two components are necessary for the function of the effector delivery system. In addition, each strain contained several *Salmonella* pathogenicity islands (SPIs), as shown in Fig. 1. Specifically, each isolate had a total of 22 SPIs, including SPI-1, SPI-2, SPI-3, SPI-4, SPI-5, SPI-9, SPI-12, SPI-13, SPI-14, and CS54_island. Additionally, C63PI was detected in the *S.* Infantis 9851 and *S.* Infantis 9916 strains (Fig. 1).

The virulence factors within mobile genetic elements (MGE) of each cgMLST cluster are listed in Table 4. The same virulence profile with 14 virulence factors in total was displayed in the mega-plasmid of each strain and involved in the adherence (*clpC*,* faeD*, and *faeE*) and yersiniabactin (*ybtE*,* ybtT*,* ybtU*,* ybtA*,* ybtQ*,* ybtX*,* ybtS*,* ybtP*,* irp1*,* irp2*, and *fyuA*). Prophages within bacterial chromosomes and plasmids were subjected to virulence gene screening (Table 4). No virulence genes were found in the prophage sequences from the plasmids. Several virulence genes were presented in the prophage sequences of bacterial chromosomes, revealing different virulence profiles among the three cgMLST clusters. In detail, the prophages of bacterial strains in clusters 1 and 2 contained 5 genes associated with adherence (*BapA*), motility (*fljA and fljB*), and metabolism (*iroB and IroC*). Additionally, the prophage sequences from two strains belonging to cluster 2 harbored the *espO1-1* gene, which encodes the type III secreted effector. Conversely, the prophage sequences within cluster 3 strains only carried 0 to 2 virulence genes listed above, and none were associated with motility and metabolism.

**Table 4:** The virulence factor carried by mobile genetic elements of *S*. Infantis strains

cgMLST Cluster	Cluster1		Cluster2						Cluster3									
Strain ID	*S.* Infantis 4897		*S.* Infantis 4900		*S.* Infantis7823		*S.* Infantis 9799		*S.* Infantis 9851		*S.* Infantis9916		*S.* Infantis 7821		*S.* Infantis 9861		*S.* Infantis 5221	
Mobile genetic elements	Plasmid	Prophages	Plasmid	Prophages	Plasmid	Prophages	Plasmid	Prophages	Plasmid	Prophages	Plasmid	Prophages	Plasmid	Prophages	Plasmid	Prophages	Plasmid	Prophages
Virulence factors	*irp1*,* irp2*,* ClpC*,* faeD*,* faeE*,* ybtA*,* ybtE*,* ybtT*,* ybtU*,* ybtP*,* ybtQ*,* ybtX*,* ybtS*,* fyuA*	*BapA*,* fljA*,* fijB*,* iroB*,* iroC*	*irp1*,* irp2*,* ClpC*,* faeD*,* faeE*,* ybtA*,* ybtE*,* ybtT*,* ybtU*,* ybtP*,* ybtQ*,* ybtX*,* ybtS*,* fyuA*	*espO1*,* BapA*,* fljA*,* fljB*,* iroB*,* iroC*	*irp1*,* irp2*,* ClpC*,* faeD*,* faeE*,* ybtA*,* ybtE*,* ybtT*,* ybtU*,* ybtP*,* ybtQ*,* ybtX*,* ybtS*,* fyuA*	*espO1*,* BapA*,* fljA*,* fljB*,* iroB*,* iroC*	*irp1*,* irp2*,* ClpC*,* faeD*,* faeE*,* ybtA*,* ybtE*,* ybtT*,* ybtU*,* ybtP*,* ybtQ*,* ybtX*,* ybtS*,* fyuA*	*BapA*,* fljA*,* fljB*,* iroB*,* iroC*	*irp1*,* irp2*,* ClpC*,* faeD*,* faeE*,* ybtA*,* ybtE*,* ybtT*,* ybtU*,* ybtP*,* ybtQ*,* ybtX*,* ybtS*,* fyuA*	nd	*irp1*,* irp2*,* ClpC*,* faeD*,* faeE*,* ybtA*,* ybtE*,* ybtT*,* ybtU*,* ybtP*,* ybtQ*,* ybtX*,* ybtS*,* fyuA*	*BapA*,* espO1*	*irp1*,* irp2*,* ClpC*,* faeD*,* faeE*,* ybtA*,* ybtE*,* ybtT*,* ybtU*,* ybtP*,* ybtQ*,* ybtX*,* ybtS*,* fyuA*	*espO1*	*irp1*,* irp2*,* ClpC*,* faeD*,* faeE*,* ybtA*,* ybtE*,* ybtT*,* ybtU*,* ybtP*,* ybtQ*,* ybtX*,* ybtS*,* fyuA*	*BapA*	*irp1*,* irp2*,* ClpC*,* faeD*,* faeE*,* ybtA*,* ybtE*,* ybtT*,* ybtU*,* ybtP*,* ybtQ*,* ybtX*,* ybtS*,* fyuA*	*espO1*
Fimbriae cassette	**+**	nd	**+**	nd	**+**	nd	**+**	nd	**+**	nd	**+**	nd	**+**	nd	**+**	nd	**+**	nd
Iron metabolism	**+**	**+**	**+**	**+**	**+**	**+**	**+**	**+**	**+**	nd	**+**	nd	**+**	nd	**+**	nd	**+**	nd
Motility	nd	**+**	nd	**+**	nd	**+**	nd	**+**	nd	nd	nd	nd	nd	nd	nd	nd	nd	nd
Adherence	**+**	**+**	**+**	**+**	**+**	**+**	**+**	**+**	**+**	nd	**+**	**+**	**+**	nd	**+**	**+**	**+**	nd

_+: the genes related to the corresponding function were detected; nd: Not detected_

### ARG profile of *S.* Infantis

**Fig. 2:**
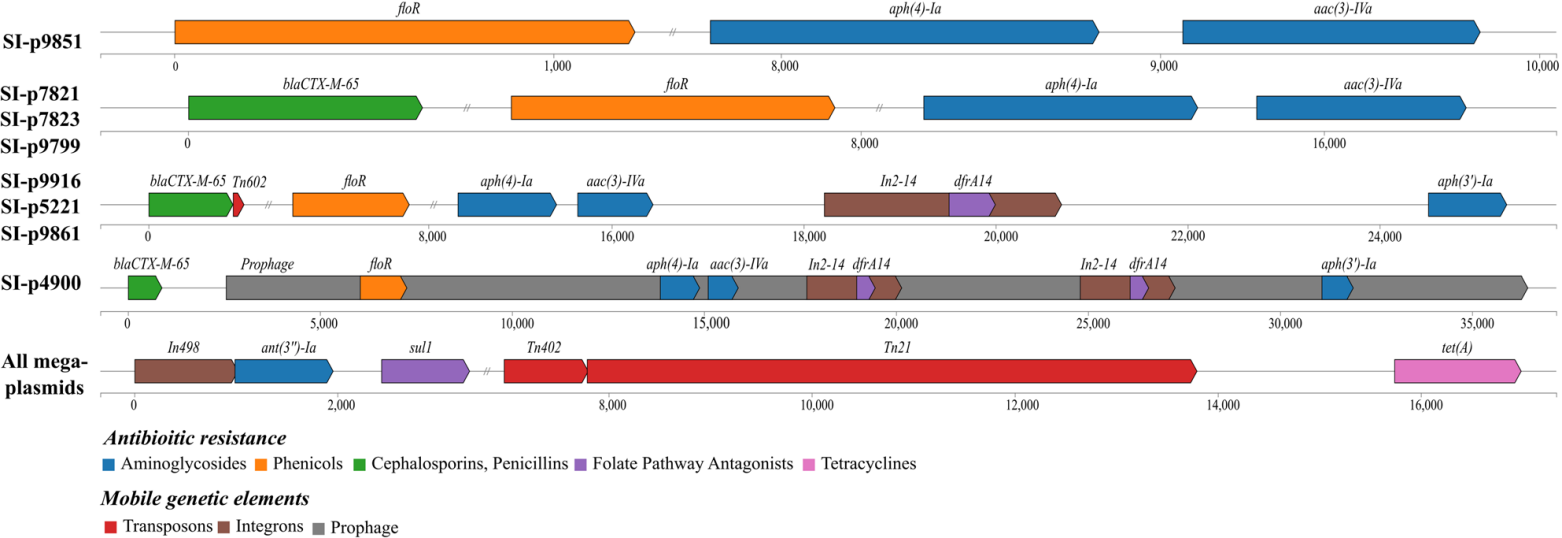
Distinct genomic context of mobile genetic elements and resistance elements within nine mega-plasmids. The ARG types and mobile genetic elements were annotated and represented in different colors

The ARG profile of *S.* Infantis indicated that five or more ARGs were detected in each strain, of which most ARGs were located in the mega-plasmids (Table 2). The chromosomes of 9 *S.* Infantis isolates contained the gene *aac(6’)-Iaa*, encoding aminoglycoside acetyltransferase. The majority of ARGs harbored by the mega-plasmids were classified into five different ARG types (Fig. 2). The plasmid of *S*. Infantis 9851 contained three ARGs, including *floR*,* aph(4)-Ia*,* and acc(3)-Iva*, while three plasmids from *S.* Infantis 7821, 7823, and 9799 had one additional *bla*_CTX−M−65_ gene. There were also several ARGs flanked by multiple mobile genetic elements, such as transposons, integrons, and prophages. For example, transposon Tn602 was adjacent to the *bla*_CTX−M−65_ gene in the plasmids of *S.* Infantis 9916, 5221, and 9861. Moreover, these three plasmids had an In2-14 integron comprising the *dfrA14* cassette and the gene *aph(3’)-Ia*. Different from other ARG types, *S.* Infantis 4900 plasmid contained 6 ARGs within an intact prophage sequence, of which two *dfrA14* genes were located within two integrons In2-14, respectively. In addition, each of the mega-plasmids carried an integron class In498, which consisted of *ant(3”)-Ia* and *sul1* genes and two transposons (Tn402 and Tn21) right next to the *tet(A)* gene.

The ARG profile of each bacterial genome, particularly those in the plasmids, was largely consistent with the phenotypic resistance profile of 9 isolates (Table 2). The gene *bla*_CTX-M-65_, identified in most *S.* Infantis strains, likely contributed to the observed resistance to cephalosporin and penicillin antibiotics. Each strain showed resistance to aminoglycosides, possibly due to the presence of *ant(3’’)-Ia* and *aac(3)-Iva* in the plasmids. Resistance to tetracycline and phenicol antibiotics, observed in most strains, likely resulted from the acquisition of the genes *tet(A)* and *floR*, respectively. However, there are also some discrepancies between ARG and phenotype resistance profiles. The gene—*aac(6’)-Iaas*—identified in each bacterial chromosome did not render antibiotic resistance to amikacin. Additionally, the mega-plasmids of 6 strains contained the *sul1* and *dfrA14* genes, but these strains did not demonstrate the corresponding resistance to folate pathway antagonists. Nevertheless, the ARG results did not reasonably explain the cefoperazone and cefazolin resistance in most MDR isolates. Overall, the majority of WGS-derived ARG profiles had concordant phenotypic traits, indicating the important role of mega-plasmids in bacterial antimicrobial resistance.

## Discussion

*S.* Infantis has been reported as an emerging foodborne pathogen closely associated with the poultry production line and has contributed to increasing foodborne outbreaks in the United States and worldwide. The evolution of bacterial pathogens plays a crucial role in epidemiology and public health. Nine strains isolated from the U.S. poultry production line across various states belonged to ST32. ST32 is a major *S.* Infantis sequence type, including strains isolated from the United States, Russia, Turkey, Italy, Switzerland, Hungary, Brazil, and Japan [25–27]. In one of our ongoing studies, the cgMLST phylogenetic analysis of 91 *S.* Infantis complete genomes collected from the NCBI database indicated that nine bacterial strains sequenced in this study fell into the same cluster amongst *S.* Infantis strains isolated from diverse countries and years. No clear correlation was observed between geographical location and cgMLST clusters. A similar result was also observed in the study by Gymoese et al., where the correlation between core-genome SNP (cgSNP) clustering and geographical location did not apply to *S.* Infantis [26]. The findings suggest that the mechanisms of other genomic elements might be involved in shaping the *S.* Infantis evaluation. To better understand the unique genomic feature differences of 9 strains, the associations amongst mobile genetic elements, virulence factors, and ARGs were further revealed in this study.

A previous study suggested that prophage diversity was closely related to bacterial genome diversity in *Salmonella* strains [28]. Therefore, the prophage profile of 9 strains was investigated in this study. In our study, the top 3 reference phages within bacterial chromosomes are *Salmonella* phage BTP1, *Enterobacteria* phage pro483, and *Salmonella* phage vB_SosS_Oslo. Another study reported that prophage Escher_MG1655, *Burkholderia* phage BcepMu, *Enterobacteria* phage P4, and *Enterobacteria* phage ES18 were primary prophages among 21 *S.* Infantis strains isolated from animals, humans, and the environment in the Netherlands, the United States, and Canada [28]. The study conducted by Gymoese et al. reported that the most common prophages—*Burkholderia* phage BcepMu, *Cronobacter sakazakii* phage GAP32, *Enterobacteria* phage P4 and Phage Gifsy-1—were present in 17% of the total number of prophages within 105 *S.* Infantis genomes; the authors also mentioned that some prophages were specific to genomic clusters [26]. Together, these findings revealed the genomic diversity of *S.* Infantis prophages; cluster-specific prophages may shape the genomic differences amongst *S.* Infantis population and could be used as a potential marker to distinguish *S.* Infantis subtypes from different sources. In addition, some prophages in other *Enterobacteria* species are genomically similar to those common prophages in *S.* Infantis, as represented above, demonstrating that these prophages are broadly present across various *Enterobacteria* inter and intra species.

In this study, the distinct differences in virulence genes located on prophage sequences between different cgMLST clusters were revealed in Table 3. Previous studies demonstrated the important role of lysogenic phages in *Salmonella* pathogenicity [29]. For example, *S.* Typhimurium utilized lysogenic phage-mediated bacteriolysis to release colicin coded by a conjugation plasmid and promote bacterial fitness against other competing microorganisms [30]. In addition, Shah et al. found evidence of a lysogenic phage Fels-2 encoding biofilm-associated proteins STM2699 in *S.* Typhimurium strain, which contributed to the biofilm formation of bacterial hosts on the surface of Caco-2 cells [31]. A similar phenomenon related to *S.* Infantis has not been reported yet; however, the findings of our study imply that the distinct virulence features of prophage sequences are closely associated with cgMLST clusters. Thus, future studies regarding the correlation between prophages carrying virulence genes and *S.* Infantis pathogenicity should be investigated.

The mega-plasmid pESI has been highly related to the antibiotic resistance, pathogenicity, and fitness of *S.* Infantis isolates [32–34]. A previous study indicated *S.* Infantis strains containing the pESI plasmids were classified as a distinct clade in the hierarchical clustering analysis compared to those without carrying the plasmid [35]. In the current study, the pESI plasmids were also present in 9 *S.* Infantis strains isolated from the poultry production line. However, *S.* Infantis 4897, belonging to cluster 1 alone, was the only strain carrying a mega-plasmid (genomic size of 286,865 bp) and a small plasmid (genome size of 33,657 bp); the other 8 strains, classified in cluster 2 and cluster 3, only contained one mega-plasmid. The small plasmids, ranging from 44 kb to 96 kb as IncX1 (*n* = 3) or IncI1 (*n* = 1), were also found in four *S.* Infantis strains isolated from raw chicken meat samples in Turkey, with one to five ARGs [27]. However, no virulence genes or ARGs were detected in the small plasmid of *S.* Infantis 4897. The genes related to bacterial fitness, such as fimbriae, iron transport system, and adherence, were found in each mega-plasmid. In contrast to the consistent virulence modules, ARG profiles vary with five different types amongst pESI plasmids in this study (Fig. 2). The ARG profiles of this study showed that primary ARGs were located on the mega-plasmid of each *S.* Infantis strain. Most mega-plasmids have two ARG regions, while the mega-plasmid of *S.* Infantis 4897 only contains one ARG region, which was found in all mega-plasmids. The common ARGs present in the pESI plasmid of this study were consistent with the ARGs reported by previous studies, including *bla*_CTX−M−65_, *aac(3)-IV*, *tetA*, *suil1*, *dfrA14*, *ant(3’’)-Ia* and *floR* [27, 36, 37]. Moreover, most identified ARGs within plasmids were observed to render bacterial resistance to the corresponding antibiotics via AMR test; the findings indicate that diverse ARGs regulated by pESI plasmids contribute to the multidrug-resistant phenotype of *S.* Infantis pathogens. Further study regarding the potential correlation between ARG profile and phenotypic antibiotic resistance can be conducted by comparing the minimum inhibitory concentration (MIC) with the antibiotics found in this study. Most importantly, the majority of ARGs were bordered by diverse mobile elements, which is consistent with the finding of close correlations between resistance genes and mobile genetic elements within IncFIB plasmid reported by Kürekci et al., [36]. However, the gene content of mega-plasmids can vary significantly due to the presence of diverse ARG segments embedded with mobile modules. Overall, pESI plasmids, which encode virulence and resistance genes, are the primary factors contributing to the different genomic features of S. Infantis strains, posing a potential risk of foodborne outbreaks and public health concerns.

Prophages have also been identified as carrying ARGs at a low percentage of 0.9%, suggesting that prophage-mediated horizontal transfer of ARGs may not be the primary transmission route among *Salmonella* strains [38]. Consistent with this finding, no ARGs were detected in the prophage genome within bacterial chromosomes and most plasmids. Only an intact prophage within *S.* Infantis 4900 mega-plasmid that contained 6 intact ARGs was detected, as shown in Fig. 2. This prophage was distinct from other prophage genomes and classified into a single clade based on the phylogenetic tree of prophages within plasmids (Figure S1). Moreover, the BLASTn results showed that this prophage was also present in the pESI plasmids of an emerging *S.* Infantis (ESI) clone with 99% nucleotide coverage and identity (data not shown). The strains from this ESI clone were isolated from retail meat products and gut samples in the United States by the National Antimicrobial Resistance Monitoring System (NARMS) [37, 39]. Tyson et al. also provided genomic evidence of substantial recombination in the pESI plasmid, resulting in the presence of 0 to 10 resistance genes. This distinct ARG profile further demonstrated the proliferation of the ESI clone in poultry products from the United States, even though all chromosomes of the ESI clone shared high genomic similarity. Further studies are necessary to unveil the potential role of prophages carrying ARGs within pESI plasmids in contributing to antibiotic resistance and transmission among the *S.* Infantis population.

Plasmid conjugation plays a key role in microbial evolution, contributing to the bacterial acquisition of new phenotypes. For example, the distribution of pESI mega-plasmid was confirmed to render multidrug resistance and virulence phenotype of *S.* Infantis strains [40]. Moreover, the transmission of pESI plasmids has been reported to occur in other Salmonella serovars and various bacterial species, including commensal *Escherichia coli* and *Lactobacillus reuteri* [40, 41]. To understand the regulation and transmission mechanism, a previous study conducted comparative analyses between the pre-emergent and clonal emergent *S.* Infantis population in Israel [32]. Their results indicated that pESI increased bacterial tolerance to metal and oxidative stress, and rendered multidrug resistance. Additionally, pESI conjugation into a plasmidless *S.* Infantis strain boosted biofilm formation, adhesion, and invasion into avian and mammalian host cells. Their findings suggested that pESI played a key role in the spread of the emergent clones of *S.* Infantis in a short period. Several genetic modules within pESI plasmids—ARGs, metal tolerance cassettes, and virulence factors—could enhance *S.* Infantis host colonization. However, the individual genetic units and their regulation within bacterial hosts and further interaction with mammalian hosts remain unknown and obligate further investigation.

## Conclusions

Overall, nine antibiotic-resistant *S.* Infantis strains isolated from the poultry production line in the United States were characterized to investigate the development of their antibiotic resistance. The results showed that most *S.* Infantis strains were resistant to five or more antibiotics. The majority of ARGs were located in the pESI mega-plasmid, carried by each strain and bordered by diverse mobile genetic elements, contributing to the bacterial phenotypic resistance profile. The findings of this study provided insight into the role of mega-plasmids on the emergence and spread of antimicrobial-resistant *S.* Infantis in the United States poultry production.

## Supplementary Information

Supplementary Material 1.

## Data Availability

The complete genomes for 9 *S*. Infantis isolates sequenced in this study have been deposited in the National Center for Biotechnology Information (NCBI) under the Bioproject accession number PRJNA1153051. The short-read sequencing data of 9 bacterial strains, previously conducted by FSIS, can also be found under the Bioproject accession number PRJNA242847.

## Abbreviations

ARG: antibiotic resistance gene
MDR: multiple-drug resistant
WGS: whole-genome sequencing
REP: recurring, emerging, and persistent
AMR: antimicrobial resistance
CLSI: Clinical and Laboratory Standards Institute
cgMLST: Core genome multi-locus sequence typing
pMLST: plasmid multi-locus sequence typing
SPI: Salmonella pathogenicity island
MGE: mobile genetic elements
