# Supplementary material for: Mobile genetic elements drive the evolution and multidrug resistance of Salmonella infantis along the United States poultry production line
Source: BMC Genomics. 2026 Mar 3;27:358. doi: 10.1186/s12864-026-12607-z (PMC13063685; doi:10.1186/s12864-026-12607-z)

Supplementary Material 1.

Supplemental Figure S1.jpg

The neighbor-joining tree of prophages predicted from nine S. Infantis genomes in this study. Bootstrap analysis was conducted using 100 replicates for reliability. Branches are colored according to clusters classified from clustal omega analysis. Bold font indicates prophages located in plasmids

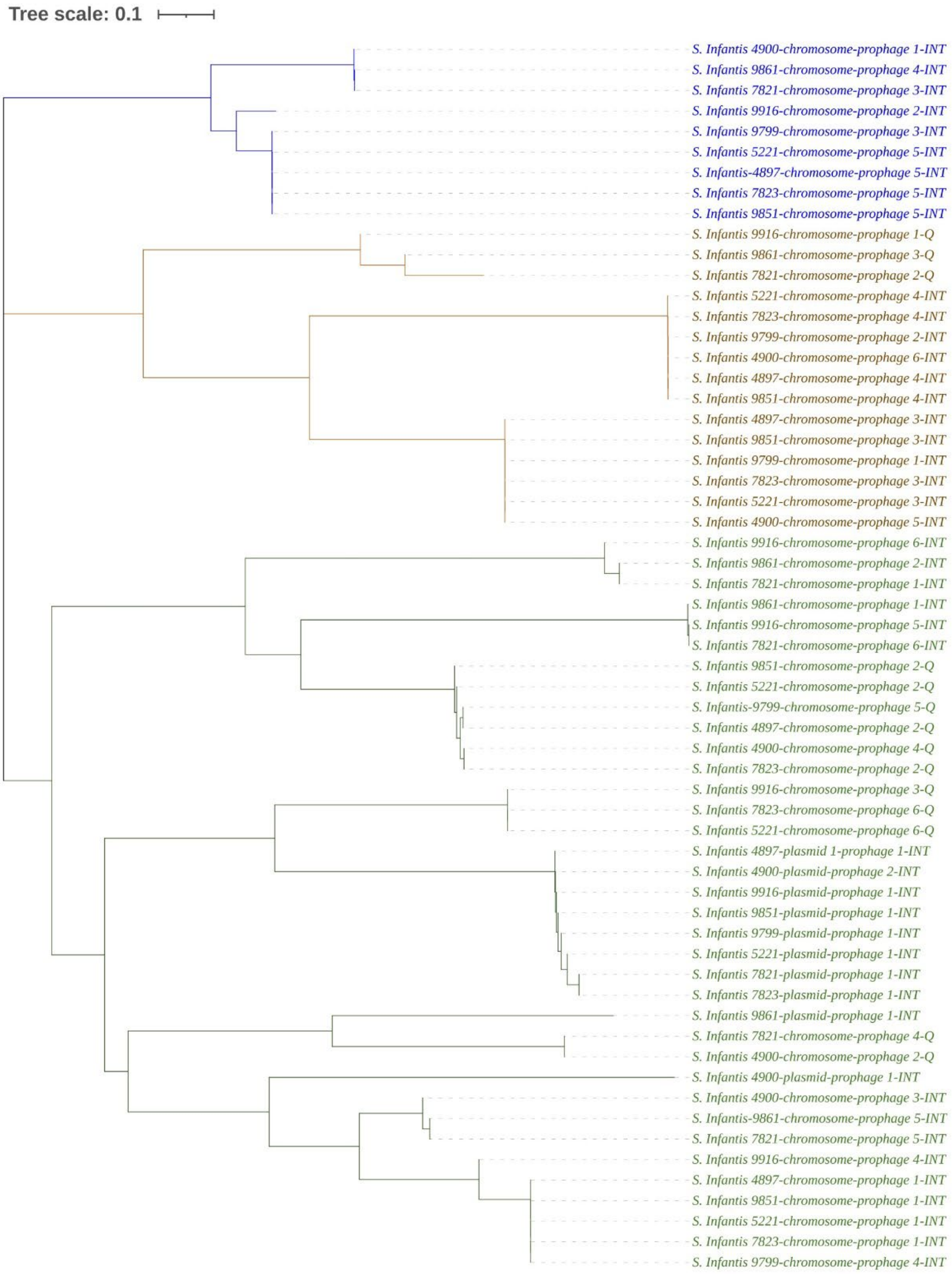

Supplement: Supplementary file 1 — Supplementary Material 1. [file 12864_2026_12607_MOESM1_ESM.pdf]
